# Supplementary material for: Restricted Water Diffusion in Diffusion-Weighted Magnetic Resonance Imaging in Pancreatic Cancer is Associated with Tumor Hypoxia
Source: Cancers (Basel). 2020 Dec 30;13(1):89. doi: 10.3390/cancers13010089 (PMC7801953; doi:10.3390/cancers13010089)
Supplement: Supplementary file 1 [file cancers-13-00089-s001.pdf]

cancers MDPI

Supplementary Materials: Restricted Water Diffusion in Diffusion-Weighted Magnetic Resonance Imaging in Pancreatic Cancer is Associated with Tumor Hypoxia

Table S1. Patient data.

Type of surgery	Loc.	Blimp-1 Allred	VEGF Allred	Tumor-stroma ratio	ADC [µm2/s]	Adjuv. chemotherapy	Follow-up (days)	Recurrence
cWhipple	Head	0	0	20:80	1.325	Yes	107	None
Left resection	Body/tail	0	0	25:75	1.337	Yes	195	None
Left resection	Body/tail	0	3	30:70	1.370	Yes	88	None
ppWhipple	Head	0	4	30:70	1.260	Yes	12	None
cWhipple	Head	0	5	35:65	1.509	Yes	1430	None
Pancreatect.	Head	0	3	30:70	1.494	No	226	None
cWhipple	Head	0	4	20:80	1.450	Yes	489	None
Pancreatect.	Body/tail	2	5	35:65	1.240	Yes	414	Hepatic metastases
ppWhipple	Head	2	5	35:65	1.451	Yes	1110	None
cWhipple	Head	2	4	30:70	1.405	Yes	13	None
ppWhipple	Head	2	0	50:50	1.366	No	128	None
Left resection	Body/tail	2	5	35:65	1.046	Yes	67	None
ppWhipple	Head	2	7	35:65	1.063	Yes	12	None
Pancreatect.	Head	2	0	15:85	1.349	No	189	None
Pancreatect.	Head	2	4	30:70	1.267	Yes	0	-
ppWhipple	Head	2	3	30:70	1.381	Yes	90	None
cWhipple	Head	2	8	30:70	0.880	Yes	39	None
Pancreatect.	Head	2	7	30:70	1.024	Yes	14	None
ppWhipple	Head	2	2	30:70	1.413	Yes	1434	Local recurrence
ppWhipple	Head	3	4	80:20	1.274	No	433	Local recurrence
Pancreatect.	Head	3	3	45:55	1.143	Yes	781	Local recurrence
ppWhipple	Head	3	4	40:60	1.297	Yes	147	Hepatic metastases
Left resection	Body/tail	3	4	45:55	1.228	Yes	222	Peritoneal metastases
Left resection	Body/tail	3	5	30:70	1.180	Yes	34	None
ppWhipple	Head	3	2	35:65	1.042	Yes	0	-
ppWhipple	Head	4	5	40:60	1.156	Yes	305	Local recurrence & pulmonary metastases
cWhipple	Head	4	2	40:60	1.204	Yes	311	Local recurrence & mesenterial lymph node metastases

cWhipple	Head	4	7	30:70	1.121	Yes	394	Local recurrence
ppWhipple	Head	4	5	20:80	1.151	Yes	0	-
ppWhipple	Head	4	5	30:70	1.242	Yes	9	None
ppWhipple	Head	4	8	70:30	1.085	Yes	0	-
cWhipple	Head	4	4	25:75	1.287	Yes	10	None
ppWhipple	Head	5	3	50:50	1.225	Yes	10	None
ppWhipple	Head	5	8	40:60	1.052	Yes	0	-
ppWhipple	Head	5	7	30:70	0.875	Yes	1988	None
Pancreatect.	Head	5	7	20:80	1.054	No	149	Hepatic metastases
Left resection	Body/tail	5	7	25:75	1.159	Yes	159	None
Left resection	Body/tail	5	3	45:55	1.122	Yes	289	Local recurrence
ppWhipple	Head	5	7	30:70	0.827	Yes	300	Local recurrence
ppWhipple	Head	6	3	35:65	1.002	No	18	None
Left resection	Body/tail	6	8	50:50	1.036	No	34	None
cWhipple	Head	7	5	50:50	0.988	Yes	302	Local recurrence

Adjuv.: adjuvant; cWhipple: classical Whipple procedure; loc.: localization; Pancreatect.: Total pancreatectomy; ppWhipple: pylorus-preserving Whipple procedure.

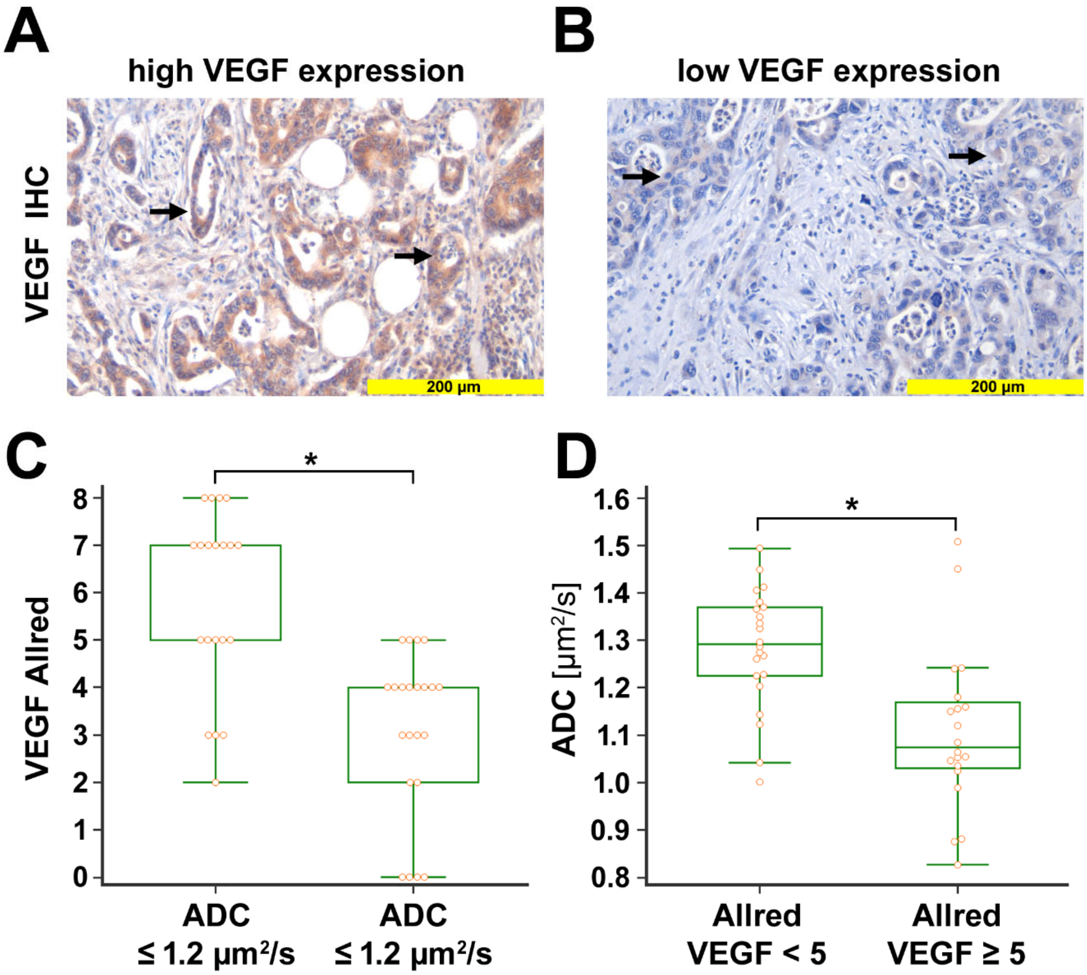

Figure S1. VEGF expression and correlation with ADC values. (**A**) Anti-VEGF immunohistochemical (IHC) staining of a pancreatic cancer tissue sample from a patient with low diffusivity (corresponding to patient 1 from Figure 1) shows strong cytoplasmatic VEGF expression in tumor cells (arrows). (**B**) Anti-VEGF IHC staining from a patient with high diffusivity (corresponding to patient 2 from Figure 1) shows weak cytoplasmatic VEGF expression in tumor cells (arrows). (**C**) Box-and-whisker plot showing the distribution of VEGF Allred scores relative to ADC values. Median VEGF Allred scores were significantly higher in patients with low ADC values (≤1.2 μm²/s) compared to patients with high ADC values (>1.2 μm²/s) (median score 7, IQR 5 to 7, versus median score 4, IQR 2 to 4; $p < 0.001$). (**D**) Box-and-whisker plot showing the distribution of ADC values relative to VEGF Allred scores. Patients with low VEGF Allred scores (<5) had significantly higher median ADC values than those with high VEGF expression (Allred scores ≥ 5) (median ADC 1.292 μm²/s, IQR 1.225 μm²/s to 1.370 μm²/s, versus 1.0741 μm²/s, IQR 1.030 μm²/s to 1.169 μm²/s; $p < 0.001$).

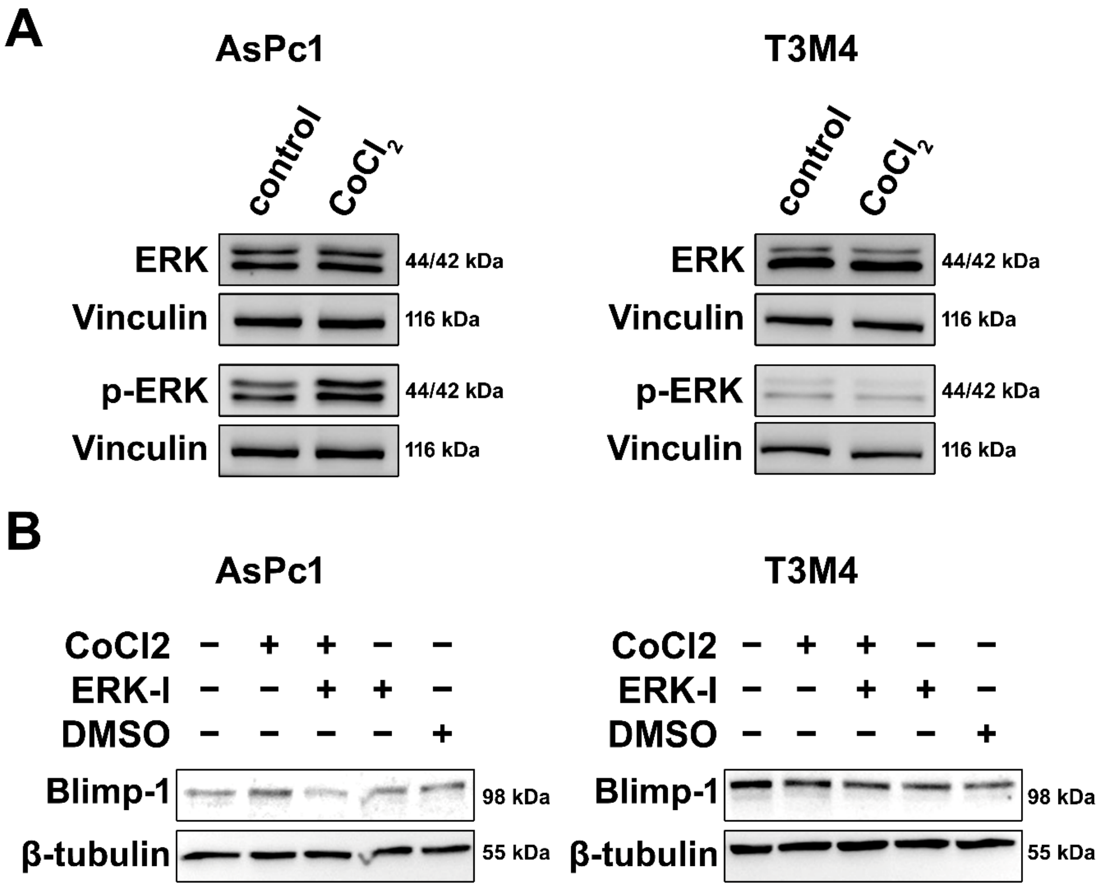

Figure S2. Possible involvement of ERK-pathway on Blimp-1 induction. (**A**) Western blots showing increased ERK phosphorylation in AsPc1 cells following treatment with $CoCl_2$ compared to vehicle control. In T3M4, phosphorylation of ERK did not differ between cells following treatment with $CoCl_2$ and vehicle control. (**B**) Addition of an ERK inhibitor (ERK-I) counteracted the increase of Blimp-1 in AsPc1 cells treated with $CoCl_2$. Again, no significant difference was seen in T3M4. These data indicate that Blimp-1 induction under hypoxic conditions could in part be dependent on ERK.

.

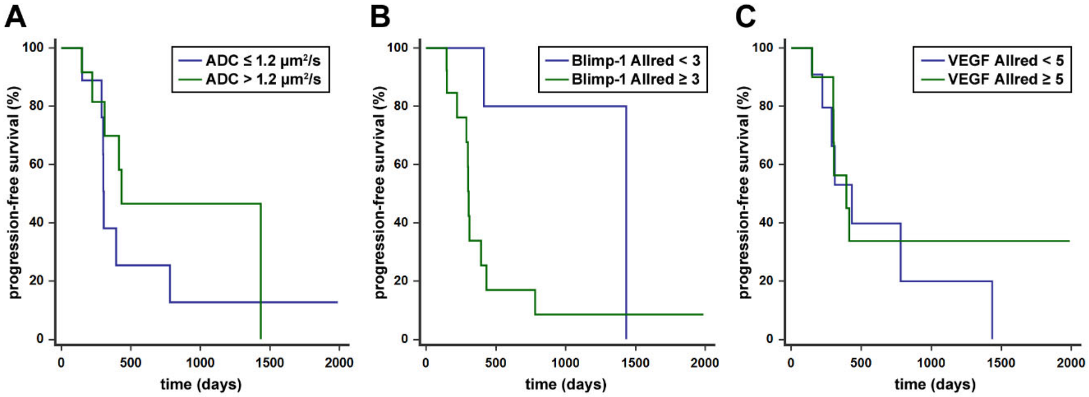

Figure S3. Progression-free survival. (**A**) We observed a trend towards shorter progression-free survival (PFS) in patients with low ADC values (≤1.2 µm²/s) compared to patients with high ADC values (>1.2 µm²/s, median 305 days versus 433 days, p = 0.282). (**B**) PFS was significantly shorter in patients with high Blimp-1 Allred scores (≥3) than in patients with low Blimp-1 Allred scores (<3, median 305 days versus 1434 days, *p* = 0.020). (**C**) PFS of patients with high VEGF Allred scores (≥5) was not significantly different from PFS of patients with high VEGF Allred scores (<5, median 394 days versus 433 days, *p* = 0.625).
